# Antifouling potential of enzymes applied to reverse osmosis membranes

**DOI:** 10.1016/j.bioflm.2023.100119

**Published:** 2023-04-01

**Authors:** Mojtaba Khani, Mads Frederik Hansen, Susanne Knøchel, Behnam Rasekh, Karim Ghasemipanah, Seyed Morteza Zamir, Mohsen Nosrati, Mette Burmølle

**Affiliations:** a Biotechnology Group, Faculty of Chemical Engineering, Tarbiat Modares University, P.O. Box, 14115-114, Tehran, Iran; b Section of Microbiology, Department of Biology, University of Copenhagen, Universitetsparken 15, DK-2100, Copenhagen, Denmark; c Section of Microbiology and Fermentation, Department of Food Science, University of Copenhagen, Rolighedsvej 26, DK-1958, Frederiksberg, Denmark; d Environment and Biotechnology Research Division, Research Institute of Petroleum Industry, P.O. Box, 14665-137, Tehran, Iran

**Keywords:** Reverse Osmosis Membrane, Biofilm formation, Water recovery, Biofouling, Enzymes, Confocal laser scanning microscopy

## Abstract

Many companies in the food industry apply reverse osmosis (RO) membranes to ensure high-quality reuse of water. Biofouling is however, a common, recalcitrant and recurring problem that blocks transport over membranes and decreases the water recovery. Microorganisms adhering to membranes may form biofilm and produce an extracellular matrix, which protects against external stress and ensures continuous attachment. Thus, various agents are tested for their ability to degrade and disperse biofilms. Here, we identified industrially relevant bacterial model communities that form biofilms on RO membranes used for treating process water before reuse. There was a marked difference in the biofilm forming capabilities of bacteria isolated from contaminated RO membranes. One species, *Raoultella ornithinolytica*, was particularly capable of forming biofilm and was included in most communities. The potential of different enzymes (Trypsin-EDTA, Proteinase K, α-Amylase, β-Mannosidase and Alginate lyase) as biofouling dispersing agents was evaluated at different concentrations (0.05 U/ml and 1.28 U/ml). Among the tested enzymes, β-Mannosidase was the only enzyme able to reduce biofilm formation significantly within 4 h of exposure at 25 °C (0.284 log reduction), and only at the high concentration. Longer exposure duration, however, resulted in significant biofilm reduction by all enzymes tested (0.459–0.717 log reduction) at both low and high concentrations. Using confocal laser scanning microscopy, we quantified the biovolume on RO membranes after treatment with two different enzyme mixtures. The application of proteinase K and β-Mannosidase significantly reduced the amount of attached biomass (43% reduction), and the combination of all five enzymes showed even stronger reducing effect (71% reduction). Overall, this study demonstrates a potential treatment strategy, using matrix-degrading enzymes for biofouled RO membranes in food processing water treatment streams. Future studies on optimization of buffer systems, temperature and other factors could facilitate cleaning operations based on enzymatic treatment extending the lifespan of membranes with a continuous flux.

## 1 Introduction

In recent years, several occasions of intense drought have occurred [[1], [2], [3]]. Future climate-change scenarios are associated with a risk of extended periods of drought leading to water scarcity [[4], [5], [6], [7]]. Access to clean water is one of the 17 sustainability goals of the United Nations [8] and it is pivotal to ensure means for water recovery for health and productivity. A promising technology is the application of reverse osmosis (RO) membranes, which has demonstrated the ability to deliver high quality water and eliminate pollutants [9]. A drawback is however membrane fouling, which limits the system level performance [[10], [11], [12]]. A major cause of biofouling on RO membranes is the accumulation of microorganisms that colonize across the membrane and eventually limit the flux [13]. The communities of microorganisms, known as biofilms, produce and accumulate extracellular biopolymers that constitute a protective matrix [[14], [15], [16]]. In many cases, multiple species will accumulate over time [17,18], which may give rise to community-intrinsic properties [19]. This also applies for biofouled RO membranes where great microbial diversity is observed [10,11]. These interspecies interactions impact biofilm formation [[20], [21], [22], [23], [24]], can enhance tolerance for various antimicrobials [25,26] and make them challenging to remove.

In many industrial settings, chlorine is routinely applied to manage contamination by microorganisms. Polyamide composite RO membranes are however damaged by chlorine products, and hence alternatives are needed. Other compounds tested have proven too expensive, toxic in large scale or ineffective in removing biofilms [13]. Inactivation of bacteria is not sufficient in the context of biofouling, since cell biomass and matrix components still present a hurdle in the filtration process [27]. The application of enzymes has previously been shown to disrupt biofilms and reduce biomass in industrial and clinical settings [[28], [29], [30], [31], [32]]. Thus, enzymes that cleave matrix components could potentially be valuable agents in the combat against biofouling on RO membranes, increase flux, save energy and ensure long time performance of the system. Recently, a novel screening platform confirmed the ability of some enzymes to outperform traditional RO membrane cleaning methods [33]. Additionally, the application of polysaccharide-degrading enzymes proved advantageous on longer terms, as removal of the polysaccharide rich matrix layer postponed refouling of new contaminant agents [34].

Here, we identified biofilm-forming communities among bacteria previously isolated from biofouled membranes to evaluate the biofilm-reducing effect of different enzymes. Subsets of the best biofilm formers were grown on RO membranes and subsequently exposed to α-Amylase, Alginate lyase, β-Mannosidase, proteinase K and Trypsin-EDTA. Our data showed that higher concentrations of enzymes had the strongest effect in terms of biofilm reduction. Longer duration of treatment did however significantly improve the effect of low concentrations enzymes, indicating a trade-off between concentration and time. Among the enzymes tested, β-Mannosidase was the most effective individual anti-fouling agent and the combination of all enzymes resulted in the strongest overall effect.

## 2 Materials & methods

### 2.1 Bacterial strains

Biofilm formation varies depending on species composition. Thus, we prioritized the use of strains previously isolated from contaminated RO membranes [11] (Table 1) as representative model strains in this study. All strains were routinely grown in TSB (VWR) or on 1.5% agar TSA plates. Five strains were selected due to relative high biofilm formation, or potential impact on communities, and further identified by PCR amplification and sequencing of the 16S rRNA encoding gene. Template DNA for PCR was achieved by mixing a single colony in 0.1 ml molecular water and subsequently the suspension was boiled at 98 °C for 10 min. PCR reactions were performed with HiFi Polymerase, 5x reaction buffer (PCRBIO), Nuclease-free water, primer 27f (5′-AGAGTTTGATCCTGGCTCAG-3′) and primer 1492r (5′-GGTTACCTTGTTACGACTT-3′). The PCR protocol included an initial denaturation step at 94 °C for 2 min, then 30 cycles of amplification (denaturation at 95 °C for 20 s, annealing at 55 °C for 40 s, extension at 72 °C for 70 s), and a final extension step at 72 °C for 7 min. Electrophoresis confirmed the presence of a single band for each reaction and the products were purified, respectively (QIAQuick PCR purification kit) and Sanger sequenced with the same primers (Eurofins). Sequence similarity was assessed by BLASTn [35] and percentage similarity and best hit is displayed in Table 1.

**Table 1:** Strains used in this study, and the sequence similarity to the curated NCBI RefSeq database for bacterial and archaeal 16S ribosomal sequences.

Number	Strain	Reference	16S Similarity	Best hit Accession no.
1	*Escherichia coli* 3bb	[11]	n.a.	–
2	*Bacillus* sp. 1C	[11]	99.42%	NR115526
3	*Pseudomonas proteolytica* 1C206	[11]	99.49%	NR025588
4	*Enterobacter* sp. 8d	[11]	n.a.	–
5	*Stenotrophomonas maltophila* W11	[11]	99.40%	NR040804
6	*Raoultella ornithinolytica* 2B	[11]	99.65%	NR044799
7	*Rothia nasimurium* 1C4	[11]	98.86%	NR025310

In order to address the colony morphology, overnight bacterial cultures were diluted to OD_600_ = 0.15 in TSB and 5 μl was then spotted on TSA plates complemented with 40 μg/ml Congo red (Direct red 28) and 20 μg/ml Coomassie brilliant blue G250. Plates were incubated at 25 °C and images were acquired at day 1, 2, 5 and 8.

### 2.2 Biofilm formation screening and effect of single enzymes

The biofilm screening was performed in 96-well plates with the application of the Nunc-TSP peg lid system (Calgary device) [36]. This assay quantifies adherence of cells and matrix to the peg surface. Overnight cultures were adjusted to OD_600_ = 0.15 in growth media and mixed in all possible combinations up to a community of four members (Equation 1). Plates were sealed with parafilm and incubated statically at 25 °C.

$$\sum_{K=1}^{4}\left(\begin{array}{c} 7\\ k \end{array}\right)=98$$

***Equation 1**. Communities of bacteria with up to four species present, and where each species maximum appear once in each combination, in a pool of seven species, result in a total number of combinations of 98. K is the binomial distribution.*

Post incubation, peg lids were washed by five successive transfers in phosphate-buffered saline (PBS) and subsequently stained in 160 μl of an aqueous 1% (w/v) Crystal Violet solution. After 20 min of staining, the lids were washed five times in PBS and then placed in a new microtiter plate with 200 μl of 96% ethanol in each well for 30 min. The dissolved stain was quantified at 590 nm in an ELx808™ Absorbance Microplate Reader (BioTek Instruments). The CV-ethanol suspension was diluted with 96% ethanol and re-measured when the OD_590_ > 2.

To address the effect of duration and concentration of different enzyme treatments, biofilms were grown using the same peg-lid system for 72 h. Three plates were prepared simultaneously with the different bacterial combinations. After incubation, biofilm formation was quantified for a single plate by crystal violet staining (N_C_), while the two others were moved to 96-well plates with different enzyme solutions prepared (Table 2). Biofilm formation was quantified for the second plate after 4 h of incubation in the presence of enzymes, while the third plate was quantified after 24 h of incubation. The effect of enzyme treatment was calculated as biofilm remaining after treatment compared to initial biofilm (log(N_T_/N_C_)) where N_T_ is the OD_590_ value from the staining procedure at time (T) = 4 h or 24 h, respectively.

**Table 2:** Enzymes used in this study and their origin.

Enzyme	Origin	Cat. No.	Supplier
Trypsin-EDTA	–	15400054	Thermo Fisher
Proteinase K	*Tritirachium album*	P2308	Sigma-Aldrich
α-Amylase	*Aspergillus oryzae*	E-ANAAM	Megazyme
β-Mannosidase	*Cellulomonas fimi*	E-BMOSCF	Megazyme
Alginate Lyase	–	A1603	Sigma-Aldrich

Trypsin-EDTA, α-Amylase and β-Mannosidase were provided in dissolved form by the manufacturer, while the remaining enzymes were received as powder and dissolved to high concentration stock solutions as recommended by manufacturer (*Proteinase K*: 20 mM Tris base pH = 8 + 3 mM CaCl. *Alginate lyase*: 20 mM Tris base pH = 8). Some enzymes are costly, so to test whether low concentrations could cause biofilm disruption, we tested two different concentrations of each enzyme; 0.05 U/ml and 1.28 U/ml. Concentrations were inspired by previous studies on enzyme activity on polysaccharide and protein substrates [37].

### 2.3 Confocal laser scanning microscopy and image analysis

**Fig. 1:**
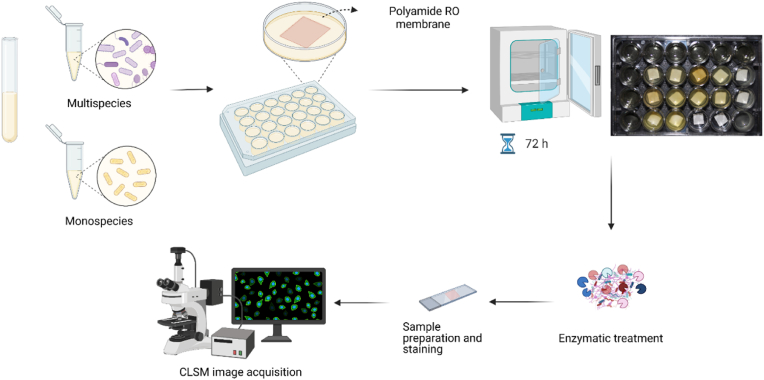
**Overall study design.** To evaluate the effect of enzyme treatments on reverse osmosis (RO) membranes, mono- or multispecies communities were cultivated in 24-well plates with the membrane present for 72 h at 25 °C. Subsequently enzymes were added, and plates were left for incubation for either 4 or 24 h, before membranes were stained with Syto9, washed with saline and imaged with an inverted confocal laser-scanning microscope (CLSM).

To facilitate formation of biofilms on RO membranes, the polyamide thin film composite RO membranes (FILMTEC™ Membranes BW30-4040) were cut into 1 cm × 1 cm pieces, thoroughly washed with sterile DI water and then stored in DI water at room temperature for 24 h. The membranes were rinsed with 70% ethanol and sterile distilled water and subsequently placed at the bottom of a 24-well microtiter plate well with the active polyamide membrane surface facing upwards (Fig. 1). Overnight cell suspensions were adjusted to an OD_600_ = 0.15 in TSB and a total volume of 1 ml of mono- or mixed cultures was added to each well. To some wells, the same volume of sterile TSB medium and water was added as control. RO-membrane patches were incubated with bacteria for 24, 48 and 72 h, respectively, at 25 °C with shaking (150 rpm). Subsequently, these patches were stained with DNA stain (10 μM SYTO9 for 20 min and washed in 0.9% NaCl) and the biovolume was visualized by confocal laser scanning microscopy (CLSM). Images were acquired on a LSM800 single point inverted CLSM (Carl Zeiss), with a 488 nm laser and a detection range of 485–550 nm. An EC Plan Neofluar 20x/0.50 M27 objective was used to visualize a field of view of 2048 px x 2048 px x 0.156 μm/px = 319.45 μm × 319.45 μm. In case of z-stack acquisition, an interval of 0.8 μm was used between slices. Membranes were transferred to a 24 × 50 mm #1.5 cover glass (ThorLabs CG15KH1) and fixed with an agar pad on top to prevent desiccation. The global biovolume of cells were quantified in Matlab using BiofilmQ and its graphical user interface [38] with the use of the Otsu method for thresholds [39] for segmentation.

### 2.4 Enzymatic effect on RO membranes

To address the effect of enzymes on membrane associated biofilms, membranes were prepared as described above and incubated for 72 h at 25 °C. The biofouled membranes, incubated for 72 h, were washed three times with PBS to ensure removal of non-adherent cells and then placed in wells with two enzyme solutions, respectively; mix A) a combination of 100 μg/ml Proteinase K and 1.28 U/ml β-mannosidase and mix B) mix A + 1.28 U/ml α-Amylase, 1.28 U/ml Alginate lyase and 0.0125% Trypsin-EDTA. Controls were placed in wells with 0.9% NaCl solution and no enzymes. After 24 h incubation, the suspensions were discarded and membranes were washed with 0.9% NaCl solution, stained and visualized by CLSM ([36]) ([37])

## 3 Results

### 3.1 Identification of strains with robust biofilm formation

**Fig. 2:**
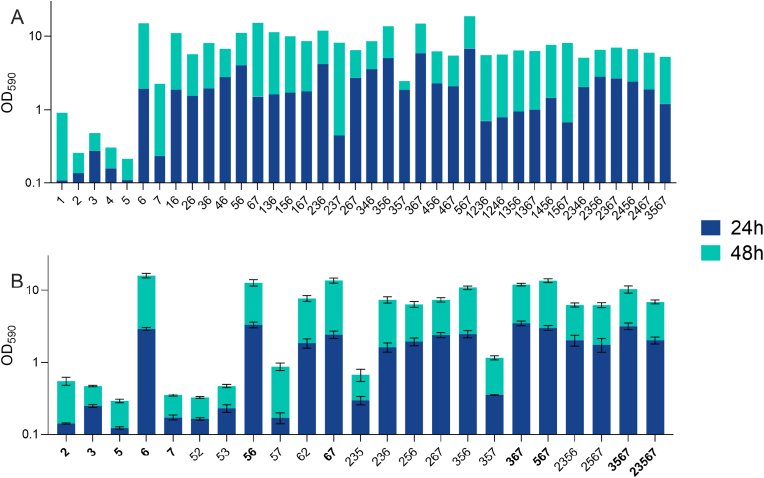
**Biofilm formation screening**. Biofilm formation was quantified after 24 h and 48 h of incubation, respectively, by crystal violet staining and subsequent optical density measurements at 590 nm. More biofilm was consistently formed after 48 h (blue + turquoise bar) than after 24 h (blue bar). **A)** A subset of the combinations with high level of biofilm formation is presented, and so are the single species measurements. All combinations can be found in Supplementary Table 1. **B)** Based on the screening, twenty-three communities of up to 5 species were selected for further analysis of biofilm dynamics. Error bars represent standard error of the mean (S.E.M.) from biological triplicates. Combinations of species in bold are those selected for further temporal analysis on RO membranes. Species numbers represent the following species: 1) *Escherichia coli*, 2) *Bacillus* sp., 3) *Pseudomonas proteolytica*, 4) *Enterobacter* sp., 5) *Stenotrophomonas maltophila*, 6) *Raoultella ornithinolytica* and 7) *Rothia nasimurium*. (For interpretation of the references to color in this figure legend, the reader is referred to the Web version of this article.)

To access the effect of enzymes on membrane-associated biofilms, a representative bacterial model community was required. An initial screening of the biofilm formation capabilities was performed on seven strains previously isolated from contaminated RO membranes (Table 1) as well as combinations up to four species. In total, 98 different bacterial communities were screened for biofilm formation (Equation 1). In contrast to previous studies on soil [21] or slaughterhouse isolates [22], no biofilm synergy among the membrane-associated bacteria was observed on PEG-lids (Fig. 2). The highest level of biofilm formation was found in communities that included *Raoultella ornithinolytica* (Fig. 2, species 6), and reached levels similar to those observed for this species in monoculture.

The seven species in the model community were all previously identified by 16S rRNA gene amplicon sequencing [11]. Based on the biofilm quantification (Fig. 2, Supplementary Table 1)) we focused on species number 2, 3, 5, 6 and 7, and verified the identity of these five strains by sequencing a longer fragment of the 16S rRNA encoding sequence. All five genera matched previous analyses, while some deviated at the species level. Specifically, species no. 3 and 7 (*P. proteolytica* and *R. nasimurium*, respectively) (Table 1), were previously identified as *P. brenneri* and *R. mucilaginosa*, respectively. In addition, sequencing of the longer fragment enabled higher resolution of the identification of species no. 5, which was identified as *S. maltophila* rather than simply *Stenotrophomonas* sp. (Table 1).

Based on the initial biofilm screening, twenty-three combinations were selected and quantified again to accommodate the stochastic nature of high-throughput crystal violet staining [40]. This verified that high-level biofilm formation was conditional on the presence of *R. ornithinolytica* (Fig. 2B, species 6), and that longer incubation (48 vs 24 h) was associated with more biofilm formation (Fig. 2).

### 3.2 Biofilm formation on RO membranes

**Fig. 3:**
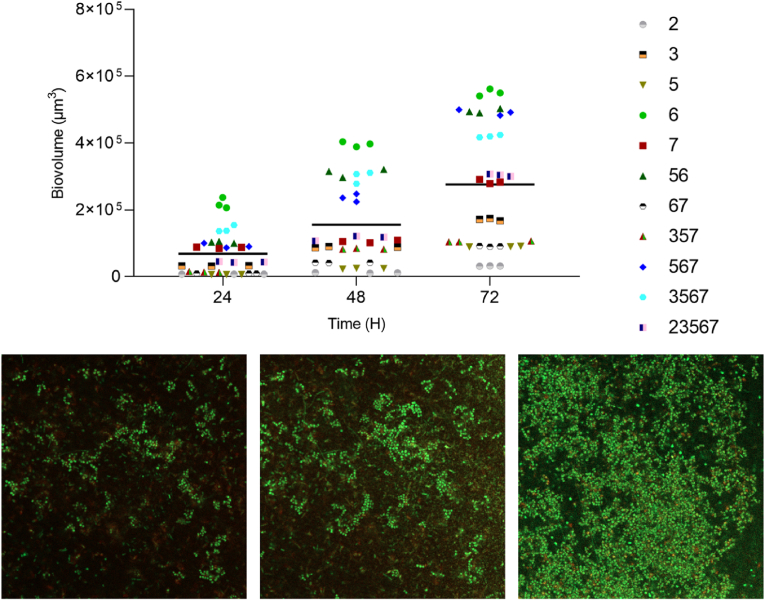
**Temporal quantification of biofilm on membranes.** Biofilm formation of eleven different species/compositions was imaged and quantified after 24, 48 and 72 h, respectively. Different symbols represent different species compositions and grand mean is represented by black line. Displayed images are biofilm formation of species 3467 at 24, 48 and 72h, respectively. Each image is 319.45 × 319.45 μm. Numbers represent the following species; 2) *Bacillus* sp., 3) *Pseudomonas proteolytica*, 5) *Stenotrophomonas maltophila*, 6) *Raoultella ornithinolytica* and 7) *Rothia nasimurium.*

Based on the screening results, six biofilm forming combinations, with relative high crystal violet values, and all five monocultures were chosen for assessment of biofilm formation on RO membranes (Fig. 2B, bold). A temporal analysis revealed that biofilm formation on RO membranes continuously increased over time (Fig. 3). Interestingly, *R. nasimurium*, which formed limited amounts of biofilm on PEG-lids (Fig. 2B, species 7), formed biofilm of relatively high biomass volume on RO membranes, reaching a volume close to the overall average (Fig. 3). Based on these analyses *Bacillus* sp. strain 1C (species 2) was excluded from subsequent experiments, as it barely formed biofilm on the membranes and did not seem to contribute with any advantages for the five species community (Fig. 3).

The production of matrix components is spatiotemporally regulated, and some are only produced at certain biofilm stages [[41], [42], [43]], meaning that the matrix is shaped by the species present and the age of the biofilm. By addition of stains that bind to proteins or polysaccharides, macro colony formation of the eleven communities was monitored on agar plates over time. In general, we initially observed relative intensive blue-stained areas in many communities, indicating reaction with the Coomassie blue. Later, colonies became intensively red, especially in the middle of colonies (Supplementary Fig. 1). Interestingly, combining strains influenced the colony morphology greatly. For example, *R. ornithinolytica* started to spatially segregate at day 2. However, when combined with *S. maltophila*, the colony developed into a condensed core with a circle of a thinner layer of cells around. Further addition of *R. nasimurium* resulted in this periphery of cells having a stronger red color, which were blue without addition of this bacterium (Supplementary Fig. 1). Although, there is a fundamental difference from growing on agar surfaces compared RO membranes submerged in liquid, these results indicated that polysaccharides and proteins were components of these strains’ matrixes both in single and mixed cultures. Thus, we selected enzymes that cleaved proteins (trypsin and proteinase K) and polysaccharides (α-Amylase, Alginate lyase and β-Mannosidase) and tested their potential as biofouling disrupting agents.

### 3.3 The biofilm disrupting potential of individual enzymes

**Fig. 4:**
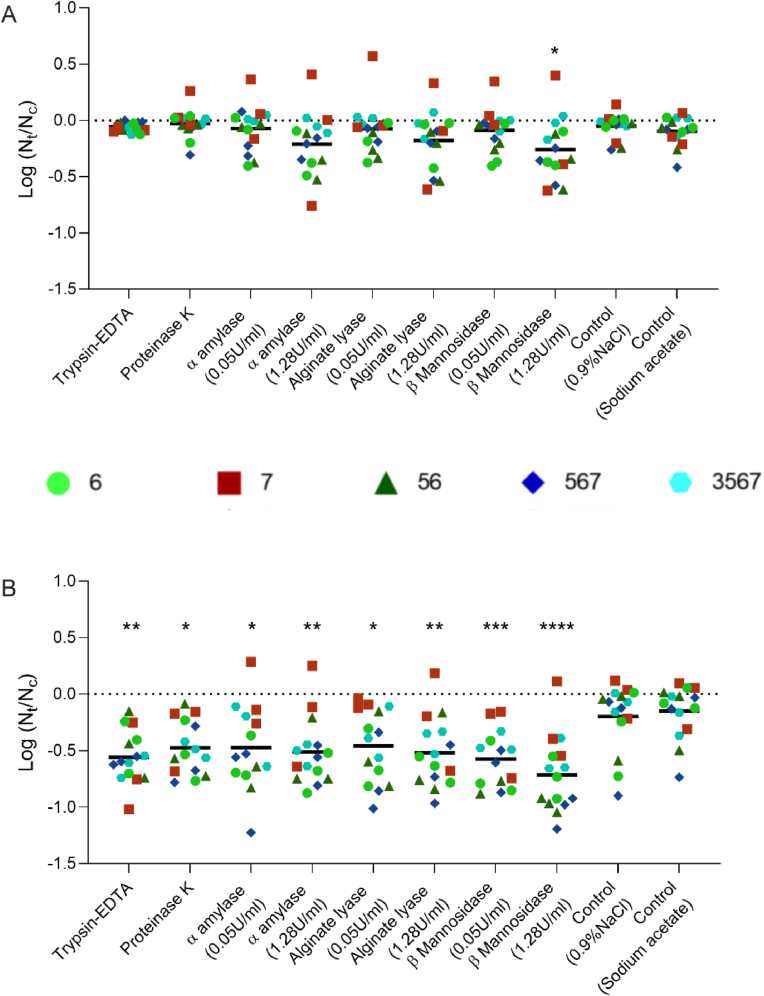
**Estimation of anti-biofilm potential of individual enzymes**. The model communities were exposed to individual enzymes for four or 24 h, respectively. Some enzymes were applied in two different concentrations (0.05 U/ml and 1.28 U/ml) to evaluate the concentration effect. Trypsin-EDTA was applied as 0.0125% solution and proteinase K at 100 μg/ml. As controls, biofilms were exposed to 0.9% saline and sodium acetate, respectively. The latter was the buffer of the α-Amylase stock. **A)** Biofilm reduction by enzymatic reduction after 4 h of enzyme exposure. Only β-mannosidase (1.28 U/ml) caused a reduction in biofilm formation within this short period of incubation. **B)** Enzymatic exposure for 24 h significantly reduced biofilm formation for all enzymes tested, independent of concentration. The higher concentration was however associated with lower P-value. Asterisks indicate P-value, *P < 0.05, **P < 0.01, ***P > 0.001 and ****P < 0.0001 (Dunnett's multiple comparison - Data points used as replicates independent of species combination). Light green circles represent biofilms of species no. 6, red squares represent species no. 7, dark green triangles represent a mix of species no. 5 + 6, blue rhombuses represent a mix of species no. 5 + 6 + 7 and cyan hexagons represent all four species together. All symbols represent one biological replicate and black lines represent the grand mean. (For interpretation of the references to color in this figure legend, the reader is referred to the Web version of this article.)

First, we addressed the potential effect by screening the individual enzymes on biofilms formed on PEG-lids. Since biofilm formation accumulated over time on membranes and reached the maximum level at the end of the experiment at 72 h (Fig. 3), we also quantified biofilm formation after 72 h in the PEG-lid assay. Again, it was evident that *R. ornithinolytica* was associated with the highest amount of biofilm formation (Supplementary Fig. 2, species 6). To address the effect of enzymes and observe a potential reduction of biofilm, a certain minimum of biofilm formation is required. Thus, we decided on a threshold for minimum biofilm formation (OD_590_ > 2.5) for evaluation of enzyme effects. The monospecies *P. proteolytica*, *S. malthophila* and the combination of these two plus *R. nasimurium* were hence excluded from the enzyme screening as their biofilm biovolumes were below the threshold (Supplementary Fig. 2). Next, the five strains/communities that produced biofilm biovolumes above the threshold were exposed to individual enzymes at different concentrations and for different treatment durations. After 4 h of treatment, only the high concentration (1.28 U/ml) of β-Mannosidase significantly reduced biofilm biovolumes compared to the saline control (Fig. 4A, average log reduction = 0.28, P < 0.05). Longer duration of treatment, however, significantly reduced biofilm formation compared to the control for all enzymes and concentrations tested (Fig. 4B). The high concentration of enzymes was associated with more biofilm reduction, with the high concentration of β-Mannosidase yielding the highest reduction (Fig. 4B, average log reduction = 0.717, P < 0.0001). The statistical analysis of enzyme effects was calculated independent of species composition. Expanding the analysis for the 24 h treatment to compare the effect of enzymes depending on combination of bacteria, we found that not all enzymes tested reduced *R. nasimurium* (species 7) biofilm biovolumes. The biofilm of this species was not significantly reduced at low concentration (0.05 U/ml) of α-Amylase and Alginate lyase (P > 0.05, Dunnett's multiple comparison). The remaining biofilm combinations were significantly reduced by all enzymes and concentrations tested compared to the controls (P < 0.05, Dunnett's multiple comparison).

### 3.4 Enzymatic effects on biofouled membranes

**Fig. 5:**
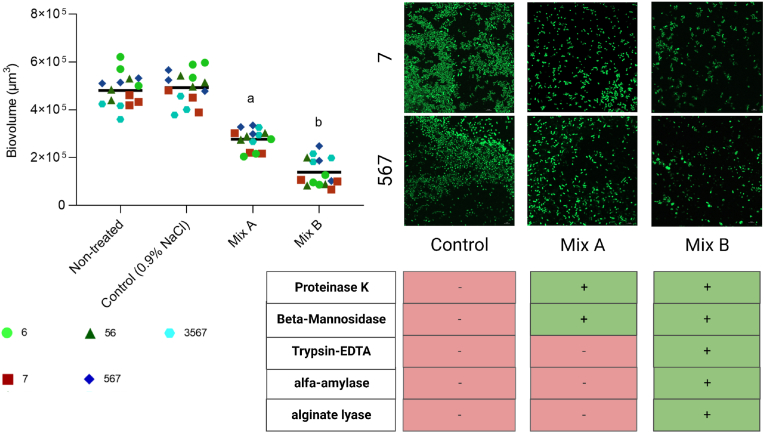
**The effect of enzyme mixtures on membranes**. The use of enzyme Mix A (100 μg/ml proteinase K + 1.28 U/ml β-Mannosidase) significantly reduced the amount of biovolume on RO membranes after 24 h of treatment compared to the non-treated starting point and the saline control (P < 0.001, Tukey's multiple comparison). The addition of more enzymes to the cocktail (Mix B: 100 μg/ml proteinase K + 1.28 U/ml β-Mannosidase + 0.0125% Trypsin-EDTA + 1.28 U/ml α-Amylase + 1.28 U/ml alginate lyase) reduced the total biovolume even further (Mix A vs. Mix B, P < 0.0001, Tukey's multiple comparison). Letters indicate significance of P < 0.001 to others letters and unmarked controls. Images display the amount of stained biofilm without treatment (control), with Mix A and mix B, respectively with species 7 and a mix of species 5, 6 and 7 as examples. Table below images indicates the presence of enzymes in specific mixtures (green = present, red = absent). Light green circles represent biofilms of species no. 6, red squares represent species no. 7, dark green triangles represent a mix of species no. 5 + 6, blue rhombuses represent a mix of species no. 5 + 6 + 7 and cyan hexagons represent all four species together. All symbols represent a biological replicate and black lines represent the grand mean. (For interpretation of the references to color in this figure legend, the reader is referred to the Web version of this article.)

After establishing that enzyme concentration and treatment duration were important factors for biofilm reduction (Fig. 4), we tested the effect of enzyme mixtures with different targets and cleavage sites on biofilms formed on RO membranes. As controls, we acquired images of membranes just before treatment start, (72 h of incubation with bacterial cultures, non-treated), and some treated with saline in parallel to the enzyme mixtures. After image acquisition, the total biovolume of each sample was quantified by image analysis. Mixture A (β-mannosidase and proteinase K) significantly reduced the biovolume present on the membranes (43%) compared to the non-treated biofilms, and addition of α-amylase, alginate lyase and trypsin-EDTA (mixture B) resulted in even further reduction (71%) (Fig. 5).

To address how much of the variation was explained by the two predictors (species composition and enzyme mixture), we performed a statistical analysis. Both predictors were identified as sources of variation and explained 3.342% and 86.36% of total variation, respectively. Further, both predictors were significant (P < 0.005 and P < 0.0001, two-way ANOVA). Comparison of the two enzymes mixtures revealed that Mix B significantly reduced biovolume compared to Mix A (Fig. 5, P < 0.0001, Tukey's multiple comparison).

## 4 Discussion

In the present study, we investigated whether enzymes removed biofilm on RO membranes. First, we characterized the biofilm formation of various combinations of species previously isolated from contaminated RO membranes. This enabled the identification of relevant model communities, and with the use of confocal laser scanning microscopy and image analysis, the effect of two different enzyme mixtures was evaluated. Biofilm disruptive capabilities were observed for the tested enzymes in an initial screen and upon exposure to mixtures of these enzymes, the biovolumes of bacteria on RO membranes were significantly reduced. Specifically, we found that a combination of proteinase K and β-Mannosidase reduced biofilm formation, and the addition of Trypsin-EDTA, α-Amylase and Alginate lyase further increased the antifouling effect.

Longer incubation was in general associated with more biofilm for all strains tested (Fig. 2). This indicates that these biofilms do not disperse by mechanisms such as quorum-sensing regulation [[44], [45], [46]] or as response to nutrient depletion [46,47]. Another study has found that removal of multispecies communities from RO membranes using traditional cleaning methods in general tended to be more challenging compared to removal of single species [48]. Although the species composition only explained 3.3% of the variation, it was a significant factor of the outcome of enzyme treatment. Combined with the observation of how mixing of species led to diverse macro colony morphologies (supplementary Fig. 1) this study emphasizes the importance of studying multispecies communities, and elucidating the community intrinsic properties that underlie such phenotypes.

The enzymes tested in this study have different targets and cleavage sites. Proteinase K is a broad-range serine protease that has previously been shown to detach and remove proteinaceous biofilms [49,50]. Trypsin is also a serine protease, routinely applied in mass spec analyses of proteins [51] and used for detachment and separation of mammalian cells [52]. EDTA functions as an ion chelating agent and enhances the effectivity of trypsin, as the target residues, on which trypsin acts, are commonly obscured by calcium and magnesium ions. Further, EDTA permeabilizes Gram-negative bacteria, releases lipopolysaccharides (LPS) [53] and functions as an antimicrobial and antibiofilm agent [54]. Since a relatively large fraction of biofilm matrixes commonly consist of exopolysaccharides [14], exopolysaccharide hydrolyzers were included. To identify relevant candidates, we searched literature and identified three enzymes previously shown effective as disruptive agents of *Pseudomonas* biofilms; α-Amylase is an α-1-4 glycoside hydrolase [[29], [30], [31]], Alginate lyase breaks the β-glycoside linkage in alginate polymers [29,30] and β-Mannosidase hydrolyzes β-1-4 linkages in polysaccharides [32].

Two enzyme mixtures were tested to elucidate the potential additive or reductive effects of enzymes. Thus, a simple mixture that included the most efficient exopolysaccharide-degrading enzyme and one of the proteases was prepared. β-Mannosidase showed the most promise as polysaccharide-cleaving hydrolase (Fig. 4) and was combined with proteinase K. Although trypsin-EDTA reduced biofilm formation marginally more than proteinase K (Fig. 4B), it was excluded from the first mixture of enzymes as some enzymes require metal ions as cofactors [55,56], and Mannosidase has previously been shown to be zinc ion dependent [57]. Thus, the chelating action of EDTA could potentially backfire and reduce efficiency of the second enzyme. The second mixture contained all five enzymes, and reduced biofilm formation significantly more effectively compared to the first mix (Fig. 5). Hence, we conclude that a simple mixture of few enzymes (in this case β-Mannosidase and Proteinase K) was useful to employ on biofouled membranes, but the application of a complex mixture with more targets and potential additive effects could further increase the removal of biofilms. This is also in line with another study reporting that a combination of cellulase and inulinase tended to remove more biofilm than the enzymes in isolation [33].

Comparison of effects of 4 vs. 24 h of enzymatic exposure indicated that increased treatment duration was associated with increased biofilm reduction (Fig. 4). Only the high concentration of β-Mannosidase reduced biofilm formation within 4 h, while all enzymes were effective after 24 h, even at low concentrations (Fig. 4). This indicates a trade-off between concentration and time; It is possible to use a low concentration of enzymes, but at the cost of longer duration of treatment, and conversely, it is likely possible to reduce treatment duration by increasing enzyme concentration. The incubation conditions may explain the lack of effect for 4 h exposure; Temperature and pH are important factors of enzyme activity [55] and it can prove challenging to find a “one size fits all” operation condition. In our experimental design, we incubated at 25 °C, at neutral pH. However, as example, the β-Mannosidase used in this study has optimal activity at 35 °C and pH = 6.5 [58], while a studied Alginate lyase originating from a *Vibrio* has its optimum at 45 °C and pH = 8.35 [59]. Thus, the sub-optimal conditions in this study could explain why the long duration of treatment is required to observe effect of all enzymes. Adjustment of pH and temperature, while treating membranes could likely increase the activity further. Although the reduction was compatible with other studies [33,34], it could possibly be improved by optimizing application conditions. It is also worth to consider subsequent flow in future assessment of enzyme effect. Enzymes do not only remove biofoulants, but also weaken the architecture of the biofilms that endure [34] and subsequent washing can be an important step in optimization of enzyme-based cleaning [33].

When comparing the effect of enzymes on individual species composition, rather than the pooled effect, we found that enzymes were effective on almost all species, which also reflects the low variance explained by species composition. The only species not affected by all enzymes after the 24 h treatment regime was *R. nasimurium* (species 7). Since this strain was the poorest of the five biofilm-formers tested (supplementary Fig. 2), it is plausible that the sensitivity of the assay was too low to detect changes. Alternatively, the matrix composition of this strain may differ from the others making it less susceptible to cleavage by the enzymes tested. Since the four other communities all included *R. ornithinolytica* (species 6), and this strain was the most potent biofilm former (Fig. 1) the enzymes seem well-suited to target the matrix of this species. This could be highly relevant as *R. ornithinolytica* is a causative agent of histamine poisoning in humans and previously classified as the same genus as the commonly known pathogen *Klebsiella* [60]. Hence, removal of this biofilm might prevent disease causing contaminations.

Overall, this study has some limitations; Experiments were conducted in a semi high-throughput manner that does not mimic real settings. Instead, this setup enabled us to screen different enzyme concentrations, durations and mixtures. We prioritized to test enzymes on relevant bacterial contaminants and the results demonstrate a potential of using enzymes as biofilm disrupting agents on RO membranes for water recovery. The current system is not limited to enzymes, but can be used for testing other agents on fouled membranes and, perhaps more importantly, the effect of combining agents. Eventually, this system could become useful for identifying conditions and agents for fouling removal on membranes, which will save both time and resources and enable efficient water recovery. This study represents a first step in the process of evaluating the use of cleaning agents on membrane-associated multispecies biofilms. Further work is needed at conditions that mimic the industrial setup with high pressure and the presence of various other contaminants. For now, it has been shown that proteases and hydrolases possess some promise, and in combination, enable removal of biofilm on membranes.

## CRediT authorship contribution statement

**Mojtaba Khani:** Conceptualization, Data curation, Investigation, Formal analysis, Methodology, Visualization, Writing – original draft. **Mads Frederik Hansen:** Data curation, Formal analysis, Investigation, Visualization, Writing – original draft. **Susanne Knøchel:** Conceptualization, Project administration, Resources, Writing – review & editing. **Behnem Rasekh:** Conceptualization, Methodology. **Karim Ghasemipanah:** Conceptualization, Methodology. **Seyed Morteza Zamir:** Conceptualization, Methodology. **Mohsen Nosrati:** Supervision, Conceptualization, Methodology, Funding acquisition. **Mette Burmølle:** Supervision, Conceptualization, Funding acquisition, Project administration, Resources, Writing – original draft.

## Declaration of competing interest

The authors declare that they have no known competing financial interests or personal relationships that could have appeared to influence the work reported in this paper.

## Data Availability

Data will be made available on request.

## Appendix A Supplementary data

Multimedia component 1 Biofilm formation of the seven model species in monoculture and combination. Biofilm was quantified by Crystal Violet staining (as described in material and methods) after 24 hours and 48 hours of incubation, respectively. Absolute CV values (subtracted by CV value of pure medium) are shown. As this was an initial screening, it was conducted in a single replicate. Some combinations were chosen for further analysis (See fig. 2B). Numbers indicate the model strains as follows: 1) Escherichia coli, 2) Bacillus sp., 3) Pseudomonas proteolytica, 4) Enterobacter sp., 5) Stenotrophomonas maltophila, 6) Raoultella ornithinolytica and 7) Rothia nasimurium. Multimedia component 1
