# Supplementary material for: Antifouling potential of enzymes applied to reverse osmosis membranes
Source: Biofilm. 2023 Apr 1;5:100119. doi: 10.1016/j.bioflm.2023.100119 (PMC10149195; doi:10.1016/j.bioflm.2023.100119)
Supplement: Multimedia component 1 — Biofilm formation of the seven model species in monoculture and combination. Biofilm was quantified by Crystal Violet staining (as described in material and methods) after 24 hours and 48 hours of incubation, respectively. Absolute CV values (subtracted by CV value of pure medium) are shown. As this was an initial screening, it was conducted in a single replicate. Some combinations were chosen for further analysis (See fig. 2B). Numbers indicate the model strains as follows: 1) Escherichia coli, 2) Bacillus sp., 3) Pseudomonas proteolytica, 4) Enterobacter sp., 5) Stenotrophomonas maltophila, 6) Raoultella ornithinolytica and 7) Rothia nasimurium. [file mmc1.docx]

| **Bacterial combination** | **24h CV value OD_590_** | **48h CV value OD_590_** |
| --- | --- | --- |
| 1 | 0.108 | 0.805 |
| 2 | 0.137 | 0.123 |
| 3 | 0.277 | 0.270 |
| 4 | 0.16 | 0.146 |
| 5 | 0.109 | 0.106 |
| 6 | 1.929 | 13.2 |
| 7 | 0.235 | 2.032 |
| 1-2 | 0.165 | 0.15 |
| 1-3 | 0.159 | 0.364 |
| 1-4 | 0.26 | 0.213 |
| 1-5 | 0.14 | 0.285 |
| 1-6 | 1.892 | 9.138 |
| 1-7 | 0.152 | 0.405 |
| 2-3 | 0.515 | 0.308 |
| 2-4 | 0.21 | 0.162 |
| 2-5 | 0.175 | 0.138 |
| 2-6 | 1.542 | 4.153 |
| 2-7 | 0.258 | 0.191 |
| 3-4 | 0.248 | 0.291 |
| 3-5 | 0.242 | 0.307 |
| 3-6 | 1.965 | 6.143 |
| 3-7 | 0.695 | 0.313 |
| 4-5 | 0.199 | 0.157 |
| 4-6 | 2.78 | 4.03 |
| 4-7 | 0.604 | 0.308 |
| 5-6 | 4.05 | 7.05 |
| 5-7 | 0.251 | 0.588 |
| 6-7 | 1.503 | 13.81 |
| 1-2-3 | 0.147 | 0.331 |
| 1-2-4 | 0.155 | 0.242 |
| 1-2-5 | 0.133 | 0.158 |
| 1-2-6 | 1.11 | 3.48 |
| 1-2-7 | 0.148 | 0.178 |
| 1-3-4 | 0.219 | 0.176 |
| 1-3-5 | 0.14 | 0.259 |
| 1-3-6 | 1.617 | 9.78 |
| 1-3-7 | 0.132 | 0.311 |
| 1-4-5 | 0.188 | 0.156 |
| 1-4-6 | 1.89 | 3.66 |
| 1-4-7 | 0.375 | 0.447 |
| 1-5-6 | 1.724 | 8.317 |
| 1-5-7 | 0.158 | 0.285 |
| 1-6-7 | 1.797 | 6.837 |
| 2-3-4 | 0.291 | 0.254 |
| 2-3-5 | 0.387 | 0.762 |
| 2-3-6 | 4.198 | 7.71 |
| 2-3-7 | 0.449 | 7.737 |
| 2-4-5 | 0.206 | 0.225 |
| 2-4-6 | 1.885 | 3.727 |
| 2-4-7 | 0.241 | 0.436 |
| 2-5-6 | 1.399 | 3.333 |
| 2-5-7 | 0.285 | 0.328 |
| 2-6-7 | 2.722 | 3.803 |
| 3-4-5 | 0.262 | 0.304 |
| 3-4-6 | 3.603 | 4.98 |
| 3-4-7 | 0.76 | 0.516 |
| 3-5-6 | 5.05 | 8.74 |
| 3-5-7 | 1.88 | 0.587 |
| 3-6-7 | 5.883 | 9.083 |
| 4-5-6 | 2.291 | 3.95 |
| 4-5-7 | 0.496 | 0.373 |
| 4-6-7 | 2.098 | 3.357 |
| 5-6-7 | 6.818 | 12.403 |
| 1-2-3-4 | 0.348 | 0.972 |
| 1-2-3-5 | 0.197 | 0.608 |
| 1-2-3-6 | 0.701 | 4.847 |
| 1-2-3-7 | 0.359 | 1.601 |
| 1-2-4-5 | 0.456 | 0.273 |
| 1-2-4-6 | 0.786 | 4.853 |
| 1-2-4-7 | 0.135 | 0.322 |
| 1-2-5-6 | 0.477 | 4.553 |
| 1-2-5-7 | 0.134 | 0.227 |
| 1-2-6-7 | 0.504 | 4.187 |
| 1-3-4-5 | 0.254 | 0.329 |
| 1-3-4-6 | 1.381 | 4.360 |
| 1-3-4-7 | 0.463 | 0.44 |
| 1-3-5-6 | 0.955 | 5.493 |
| 1-3-5-7 | 0.205 | 0.375 |
| 1-3-6-7 | 1.007 | 5.333 |
| 1-4-5-6 | 1.446 | 6.24 |
| 1-4-5-7 | 0.623 | 0.594 |
| 1-4-6-7 | 1.063 | 4.787 |
| 1-5-6-7 | 0.674 | 7.47 |
| 2-3-4-5 | 0.392 | 0.194 |
| 2-3-4-6 | 2.04 | 3.073 |
| 2-3-4-7 | 0.236 | 0.244 |
| 2-3-5-6 | 2.807 | 3.78 |
| 2-3-5-7 | 0.396 | 0.56 |
| 2-3-6-7 | 2.663 | 4.407 |
| 2-4-5-6 | 2.425 | 4.313 |
| 2-4-5-7 | 0.177 | 0.484 |
| 2-4-6-7 | 1.897 | 4.063 |
| 2-5-6-7 | 1.185 | 4.55 |
| 3-4-5-6 | 0.939 | 4.06 |
| 3-4-5-7 | 0.261 | 0.373 |
| 3-4-6-7 | 1.292 | 4.123 |
| 3-5-6-7 | 1.202 | 4.063 |
| 4-5-6-7 | 1.106 | 4.780 |
