# Supplementary material for: Antifouling potential of enzymes applied to reverse osmosis membranes
Source: Biofilm. 2023 Apr 1;5:100119. doi: 10.1016/j.bioflm.2023.100119 (PMC10149195; doi:10.1016/j.bioflm.2023.100119)
Supplement: Multimedia component 2 [file mmc2.docx]

**Supplementary Figures**

**
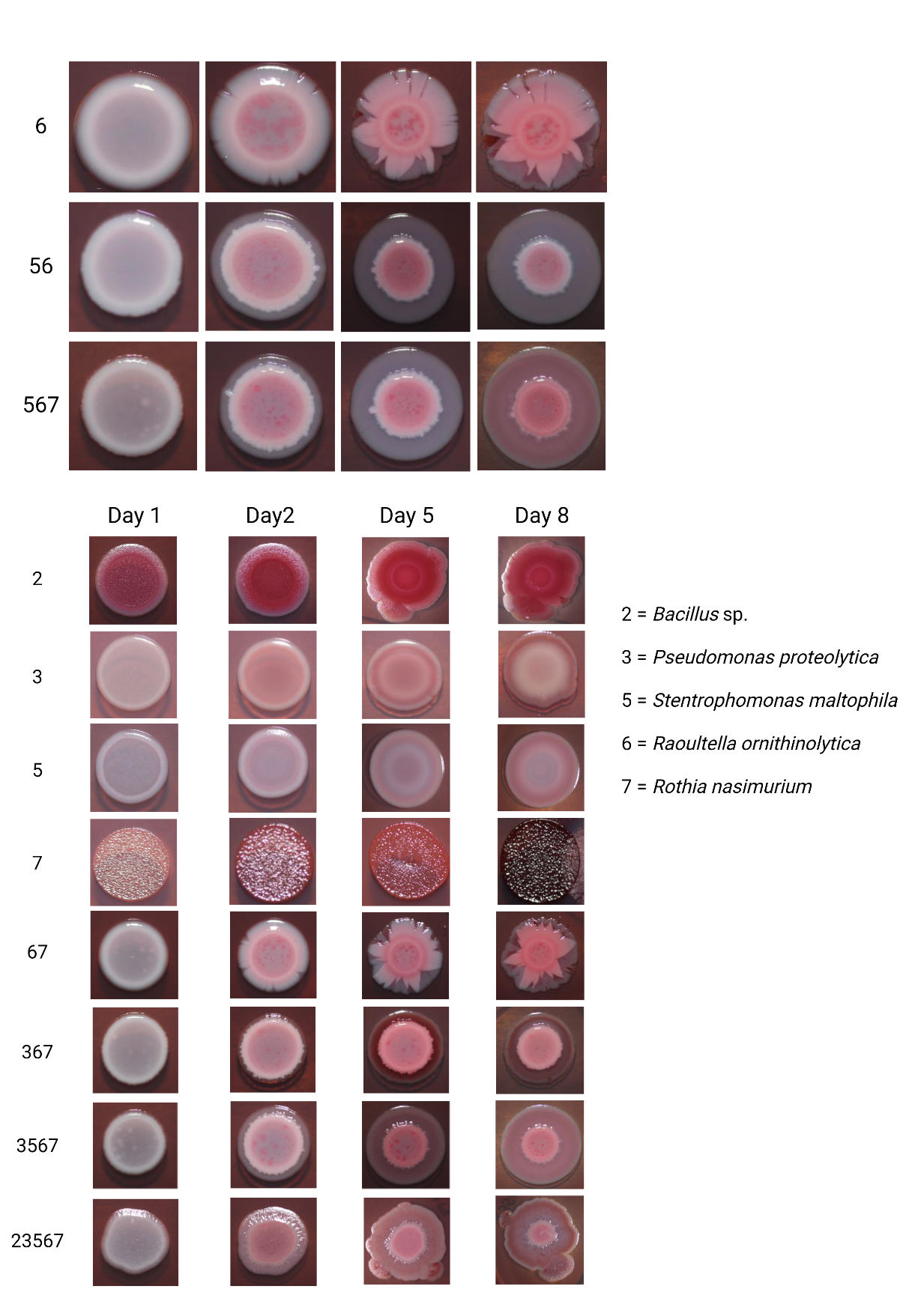
**

**Supplementary figure 1. Temporal macro colony development of single strains and multispecies combinations.** Colonies were grown on TSA supplemented with 40 µg/ml Congo red (direct red 28) and 20 µg/ml Coomasie Blue to visualize production of polysaccharides and proteins, respectively. Colonies were incubated at 25º C and images were acquired at day 1, 2, 5 and 8. Numbers on the left indicate species composition.


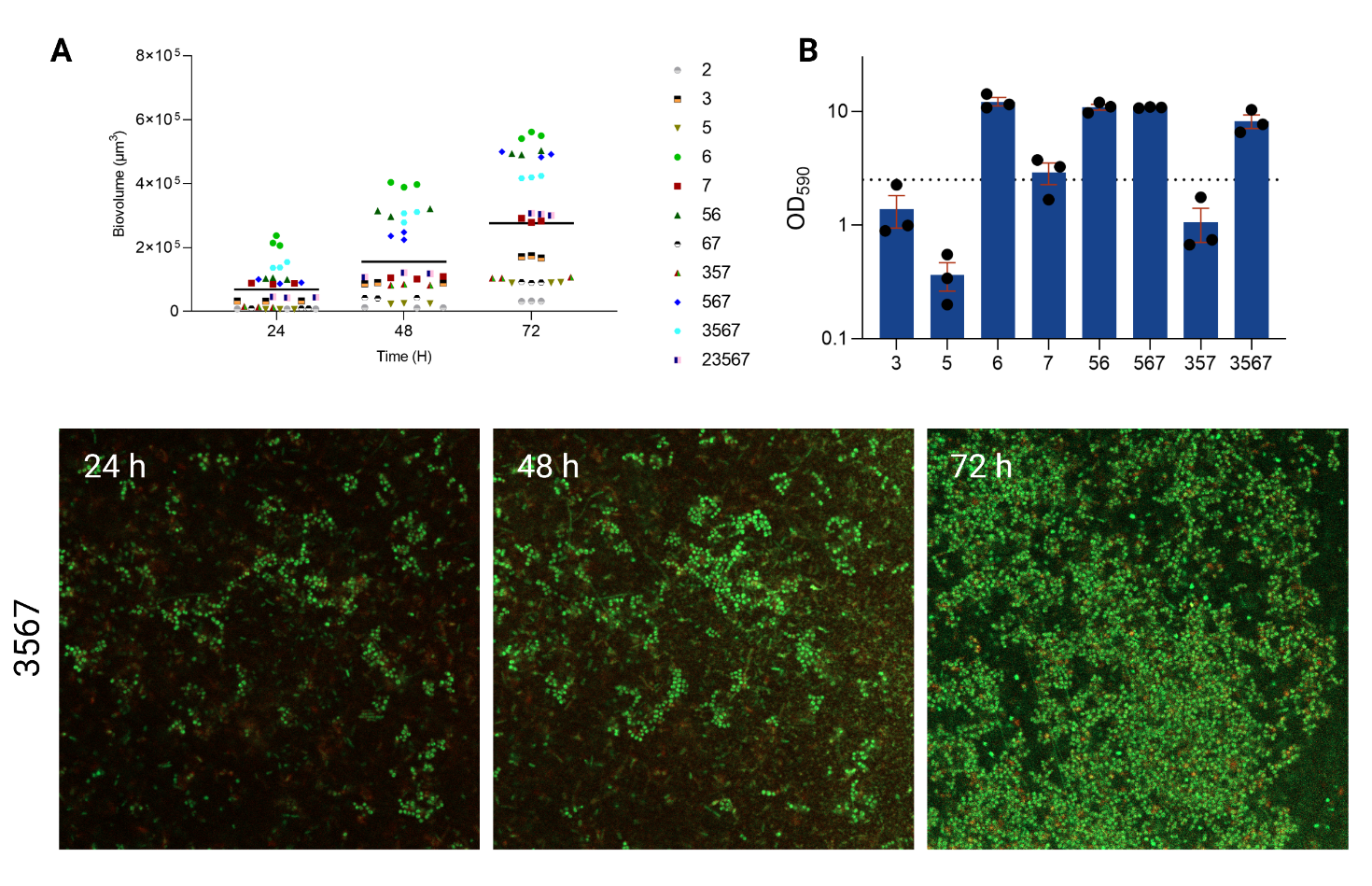


**Supplementary figure 2. Crystal violet quantification of biofilm formation after 72 hours.** Five model communities that formed biofilm above the threshold level at OD_590_ = 2.5 (dotted line) after 72h of incubation were identified and used for future experiments. Black dots represent biological replicates and red error bars represent S.E.M.
